# Abstracts and Gleanings

**Published:** 1886-04-20

**Authors:** 


### ABSTRACTS AND GLEANINGS.

**Napthalin in Diarrhœa and Typhoid Fever.**—(New York Medical Record) Attention was first strongly called to the drug by Professor Rossbach, of Sena, who at the Congress for Internal Medicine of 1884, strongly recommended it in disorders of the intestine. In chronic diarrhœa and dysentery, with or without ulcerations, Rossbach found napthaline very efficient. He gave it in doses of gr. ii to gr. x. t. i. d., increasing this sometimes to daily doses of 5 grammes. His prescription for an adult is :

℞. Naphthæ puriss.} aa ..................5.00 grammes,

Sacchar. alb., }

Olei bergamot........................0.03 grammes.

M. Sig. Pulv., div. in chart No. xx. Sig. 5 to 20 daily.

The administration of napthalin causes the fæces to be perfectly disinfected and odorless. Its action in cases of chronic diarrhœa is rapid, and most cases, if uncomplicated, are cured in from five to fifteen days. Dr. G. V. Liebig, of Reichenhall, subsequently reported a good result from enemata of napthalin in a case of dysentery (Bayr. arztl., Intell. Bl. xxxi. 29, 1884.)

Several observers have found that naphthalin given in large doses will, at times, cause a slight bladder catarrh, burning urine, and even strangurv, but Rossbach contends that these accidents can be avoided by beginning with small doses and gradually increasing them.

It is in connection with typhoid fever that the claims for naphthalin are greatest. Rossbach and his assistant, Dr. Gotze, believe that it has the power of shortening the course of this disease, lowering the temperature, and, if given early, of even aborting an attack. The Therapeutic Gazette gives the following account of Gotze's observations, made upon twenty-five cases:

"All cases, with the exception of three, were uninterruptedly treated with naphthaline during the entire typhid process, all other medication being excluded. The administration dose was 1 gramme (15 grains) and 5 to 7 grammes *pro die.* (The drug used was, according to Rossbach's recommendation, always the purissimum resublimatum, with the oil of bergamot.) In the course of the disease all patients had used over 70 grammes (2⅓ ounces), some even as much as 150 grammes (4½ ounces).

"Untoward secondary effect was noted only in a single case, in which toxic symptoms, marked by cerebral functional depression, set in. The general course of all cases was exceptionally favorable, and the benign character of the intestinal symptoms very pronounced. In three cases an actual abortion of the affection appeared unquestionable ; other cases coming under treatment early terminated in six to ten days, in others the fever did not last beyond sixteen days ; while in another series the duration of the disease was not shortened at all, though the period of thermal elevation was sensibly shortened. Three cases perished from grave complication, though not from the typhoid process itself. Of considerable interest are the comparative trials with antipyrine and naphthalin. Repeated observations convinced Gotze that naphthalin promptly reduce the febrile temperature in cases in which antipyrine (given in antifebrile does) proved useless."

**Carbolic Acid for Phthisis.**—Dr. Filleau (Buffalo Medical Journal) says: Being a firm believer in the parasitic origin of phthisis, he searched for an antiseptic or germicide which could be injected into the blood without harm to the patient. Iodoform was tried, and failed. But chemically pure carbolic acid seemed to answer all requirements. It is easily mixable with water, can be injected without pain, and never causes abscess or phlegmosis. Moreover, it has been demonstrated by Paul Bert that carbolic acid is eliminated by the lungs as well as by the kidneys, thus reaching the favorite seat of the bacilli, and acting like an antiseptic lotion. It was further observed that the bacillus tuberculosis was quickly destroyed by very weak solutions of this antiseptic.

The formula for the preparation of Dr. Filleau's solution is:

℞. Acid, carbolic, c. p. . . . . . . . . . . . . . . . . . . . . . . . . . . . . . 1 pt,
Glycerini puri . . . . . . . . . . . . . . . . . . . . . . . . . . . . . . . . 4 pts,
Aq., distillat . . . . . . . . . . . . . . . . . . . . . . . . . . . . . . . . . 94 pts.

M. Sig.—100 minims once a day, or every other day, according to the case.

The carbolic acid must be perfectly pure. That having a rose color should never be employed. The treatment should be continued persistently unless symptoms of intoxication appear, in which case the medication should be dropped.

Dr. Filleau has employed this treatment with very satisfactory results for two years. Several of his patients have been exhibited at the Hospital Cochin to the profession. He summarizes his theory and treatment as follows:

1. The parasitic origin of tuberculosis being admitted, carbolic acid, c. p., must be considered the best antiseptic to be employed in the treatment of tubercular diseases.

2. Carbolic acid is the only germicide which can be administered subcutaneously for a long time without danger.

3. The safety and toleration of carbolic acid given hypodermically have been thoroughly demonstrated.

4. By means of this treatment, the general condition of the patient is rapidly improved, and the local lesions are favorably modified.

5. The treatment in every case should be persistently carried out.

**Dr. K. I. Graves**, in the Chicago Medical Times, writes: I have found that bathing the gums at intervals of half an hour with a four per cent. solution of cocaine served to arrest diarrhœa in children in whom this symptom was evidently due to irritation from teething.

**Treatment of Pneumonia.**—A writer in Medical World says: I have had a good many practical hints from your valuable journal, and think it not amniss to exchange thoughts with my medical brethren; and as pneumonia is a prevalent disease at this season of the year throughout the country, and often fatal, I will give the treatment which I have found eminently successful, having rarely lost a case for some years past.

If called in the early stages, I at once commence with the tincture of veratrum viride or aconite, from 1 to 3 drops every hour, until temperature is reduced and pulse brought near the normal standard, and in this malarious district, a 10 grain dose of calomel with ¼ grain of podophillin. This also keeps down the temperature. In a day or two I commence with my chloride of ammonia mixture, which is as follows:

| | | |
|---|---|---|
| ℞. | Ammon. muriat | ʒ ij, |
| | Potass. chlorat | ʒ j, |
| | Syr. senegæ | ℥ j, |
| | Chloroformi | ʒ j, |
| | Aquæ comphoræ | ℥ iv, |
| | Pulv. ext. glycyrrizæ | ʒ j. |

Mix Sig. Tablespoonful every hour or two.

In this I sometimes add carb. ammonia. Here we have a defibrinating antiphlogistic and resolvent in the ammonia, a refrigerant and alterative to the mucous membranes, and at the same time organizing agent to the partially discarbonized blood in the chlorate of potassium; in the senegæ and licorice an expectorant and demulcent, and at the same time a nervous sedative; the chloroform checks the cough and quiets the system. This mixture I continue throughout, giving milk toddy as occasion requires. Locally I use hot mush poultices to the affected side in the beginning, followed in a day or two with a large blister. I also manage to get from 12 to 15 grains of quinine into the patient during the remission that generally occurs at morning. If called several days after the attack, I commence with the ammonium mixture at once.

The above treatment will cure nine-tenths of the cases. Of course I vary the treatment according to the condition of the patient, idiosyncrasies and complications. No one prescription can be invariably followed; we cannot practice physic successfully by prescriptions from books in all cases, but must be varied according to the judgment of the practitioner. Ergot has been highly lauded, and I think it very useful in congestion of the lungs; but it cannot take the place of other remedies in active inflammation of these organs.

**For Dyspepsia.**—Medical World: Five to 10 minims of glycerine of carbolic acid in a little water, after meals, is an admirable remedy for dyspepsia, and for the impaired digestion of tea-drinkers and tobacco chewers. Especially is this useful (in smaller doses) in the dyspepsia of children, associated with the presence of worms in the alimentary canal. Glycerine is in itself an anthelmintic of much power.

**Infection by Catgut Sutures.**—(Indiana Medical Journal) In a paper recently read before the Medical Society of the County of Kings, N. Y., and published in the N. Y. Medical Journal of Feb. 27, 1886, Dr. F. W. Wunderlich calls attention to the danger of infection by catgut sutures, which has not hitherto received the attention it deserves. The doctor related a case in point of carcinoma of the left breast, in which amputation was performed. The greater part of the sutures of black iron-dyed silk, and, for comparison, the inner part of the wound with eight catgut sutures. Four days afterward the dressing was removed because the patient complained of pain in the wound. The portion of the wound sutured with silk looked well, but around each catgut suture the skin was red and the epidermis was raised to a small vesicle filled with purulent serum. An attempt was made to remove the catgut sutures, but they tore, and the part imbedded in the tissues remained. Apparently there was no union along the entire line of incision. The ulceration extended quite rapidly around each catgut suture, the epidermis being raised first by purulent serum and the skin destroyed subsequently. The destructive action continued until a series of conical depressions were formed, that finally blended together, and led to the destruction of the greater portion of flaps where they had been sutured with silk healed by first intention. After elimination of the infective material, the portion sutured with catgut healed slowly by granulation. The catgut was manufactured in England, prepared by Mr. Lister's method, and kept in carbolized oil. Dr. Wunderlich believes that the only cause of the destruction of a portion of the flaps was infectious material contained in the catgut. He refers to a case reported by Prof. Sweifel, of Erlangen, in 1879, in which catgut was used to occlude a small fistula. Pyemia developed and the patient died. No other course of infection could be detected.

**Mother's Mark.**—Dr. Amundson says (in Medical Age): We all hear of, read about, and even sometimes see children marked by their mothers. While some of these cases are not well-marked, here is a well-marked case, or badly-marked—just as the reader takes it:

Mrs. B. D., living near K., Minn., while between four and five months pregnant, saw a dog attack a pig in the yard, the dog tearing off completely the pig's left ear. Four months later Mrs. B. gave birth to a daughter, now fourteen years old and presenting the following anomalies of the left side of the face: Complete absence of lobe of the ear, there being only a small mole posterior to meatus; meatus normal and hearing good. Complete paralysis of facialis sinister, with, of course, the usual symptoms. Complete paralysis of abducens oculi, hence permanent inward strabismus. The part of cornea not covered by eyelid during sleep, the seat of keratitis. Speech normal.

**Strength of Carbolic Acid Solution for Injection into Hemorrhoids.**—Dr. Kelsey (in N. Y. Medical Journal) writes: The strength of the solution must be regulated by the nature of the case, and in my own practice varies from 5 per cent. to the pure crystalized acid. In a large, vascular, prolapsing tumor, which is well defined and somewhat pedunculated, 5 drops of pure acid may be used with the expectation of producing a circumscribed slough, which will result in a radical cure. A 33 per cent. solution under the same conditions will probably produce consolidation and shrinkage without a slough, but the injections will have to be repeated several times. A small tumor which protrudes but slightly, is not pedunculated, and can be seen and felt as a mere prominence on the mucous membrane, may be cured by a single injection of a 5 per cent. solution, which will cause it to become hard and decidedly reduce its size, while an injection of a 50 per cent. solution might make considerable trouble, the remedy being too powerful for the disease. Guided by this principle, some experience will soon determine the choice of the solution. There is no arbitrary rule which can be applied to every case. As in any other surgical operation, some cases will be more satisfactory than others, and an occasional accident must be expected; but, on the whole, it seems to be the best method of treatment yet devised.

**Induction of Premature Labor.**—(Indianapolis Med. Journal) Dr. T. J. Gaillard Thomas, in a lecture published in the Physician and Surgeon, recommends the following method for the induction of premature labor. The patient is placed across the bed, with the buttocks resting near the edge, and under her is arranged a large piece of rubber oil-cloth in such a way as to drain into a tub on the floor, in which is one or two gallons water at a temperature of 98° F. The knees of the patient being properly supported, a syringe with a nozzle is carried as far into the cervical canal as it will go, and a steady stream of water is directed against the membranes. When dilatation to the extent of a half dollar is completed, which will be in the course of ten minutes, a gum catheter is inserted between the membranes and uterine walls, the patient is put to bed, and the labor allowed to proceed naturally. Dr. Thomas says this operation constitutes one of the greatest advances that has ever been made in the obstetric art, and that it is no mean triumph to be able thus to preserve a human life, which, without its aid, would have been inevitably lost. He says he can point to two dozen children in New York City whose lives were saved by this operation.

**A Strong Argument in Favor of Vaccination.**—A writer (in Medical Record) says: Among the many queries which the present extensive revival of vaccination has raised, is the one relating to the effect of vaccination upon one who has already had small-pox or varioloid. We have been somewhat surprised to find that vaccination "takes" with those who have had small-pox, two or three such cases having come under his notice. Upon inquiring of a physician, whose position at the Board of Health has given him a wide opportunity for observation, he assured us without hestitation, that after small-pox vaccination will take always, and in the primary form. Moreover, that vaccination is a surer safeguard from small-pox than the small-pox itself, for he knew of instances where unvaccinated individuals had had the disease two or three times. This information is, therefore, of great importance, for most people who have had small-pox feel they are sealed with an immunity greater than a life time of continued vaccination could purchase for them.

**Stomatitis Materna, or Nurses Sore Mouth.**—Dr. C. B. Johnson (in Practitioner and News) concludes an article on the subject as follows :

1. It is a disease to which certain nursing or pregnant women are subject.
2. It is probably true that the disease has been comparatively infrequent of late years.
3. It is *positively* true that there has been nothing, or next to nothing, in the medical literature of the past twenty years upon the subject.
4. In consequence of this many medical men of ten, fifteen, or twenty years' experience are not cognizant of ever having seen a case—would, perhaps, not recognize one if met.
5. Of the real nature of the disease, nothing is known, even by those who have made it a special study, except that it is a peculiar cachexia to which certain pregnant or nursing women are obnoxious.
6. The alimentary canal is the favorite seat of the disease ; but, should any nursing or pregnant female suffer from obscure trouble about the lungs, nose, head, or ears, the possibility of "stomatitis materna" acting as the underlying cause should be carefully considered.
7. When the disorder attends lactation, the remedy above all others is suppression of the secretion of milk.
8. When pregnancy is complicated by the disease, cases of such grave character may come up even to justify bringing on premature labors.

**Opium in Infantile Diarrhœa.**—At a recent discussion on infantile diarrhœa by the Glascow Medical Society ("Glascow Med. Jour.," Feb., 1886,) Dr. Duncan said that it required experience to give opiates to children, even in small doses; he preferred the liquid form on account of the equable diffusion of the opium, and he used the tincture, a drop for each year of the child's age. Even at that it had to be watched carefully.

**The Pharmaceutical and Therapeutic Use of Algin.**—(Physician and Surgeon, January, 1886) Mr. Watson Smith calls the attention of pharmacists and physicians to a new product, algin, discovered by Stanford. It is a residue product of the wet way of obtaining iodine from certain marine algæ, being a nitro genous principle, closely resembling albumen except in not being coagulated by heat. It forms perfectly definite alginates with metals. The alkaline alginates are soluble, but the others are insoluble. The double alginates are easy of preparation, and almost all of them are soluble. Besides possessing many curious chemical properties, giving promise of extensive applicability in pharmaceutical and other processes, algin has a notable alimentary value, and may be advantageously substituted for gum arabic in the preparation of a great number of dietetic and medicinal products.

**Placenta Prævia.**—A writer (in Iowa Medical Reporter) reports three cases of placenta Pævia as follows: The first occurred in early medical life, and its remembrance is vivid yet. The lady was a robust American, twenty-four years of age, enceinte with her first child. Repeated and profuse hemmorrhages gave unquestionable notice of its presence and danger. The case went to term. Living near, I saw the patient within ten minutes after labor commenced, and found her deluged in blood. Without a moment's delay, I introduced my hand, rapidly tore away the placenta and removed it—possibly a questionable procedure ; then grasped the feet, turned the child, and delivered the mother of a fine boy, now a prosperous farmer in California.

My second and third cases resemble my first, except that in the second I tore an opening in the placenta, through which I introduced my hand, seized the feet, turned and delivered as in case No. 1 ; while in the third I succeeded in removing the placenta at its edge. Folding it back, but failing to bring the head forward to check the hemmorrhage, I introduced my hand, seized the feet, turned and delivered as in other cases. I cannot but congratulate myself that I have never lost either mother or child in placenta prævia simple ; a result, in my opinion, of rapid work. Such obstetrics may smack of butchery, yet, life has been the happy result. Nor can I banish a suspicion that many valuable lives have been sacrificed by the timid accoucheur. These cases are, of course, extreme ones, where delay indicated immediate death. When delay appeared safe, my course has always been, call an assitant when practicable, administer an anæsthetic, and proceed to dilate and deliver in a manner more secundum artem, perhaps.

**The Use of Antipyrine During Pregnancy.**—(New York Medical Record) Professor Chiara ("Ann. di ostet."; "Repert univers. d'obstet. et de gynec.," Jan. 10–25, 1886) draws the following conclusions from his use of antipyrine in the cases of twelve pregnant women, five of whom had gone to term: 1. In therapeutic doses it has no particular oxytocic action. 2. Pregnancy, whether normal or pathological, does not modify its action. 3. It may be used with perfect safety as an antipyretic during pregnancy. 4. Like other antipyretics, it may tend to prevent abortion, an excessively high temperature being a frequent cause of that accident.

**For Inflamed Prostate.**—(Medical World) Acute inflammation and enlargement of the prostate is much relieved by the continued application of hot water to the perineum, and its injection into the rectum, giving directions for its retention. The pain often subsides in a very short time, and micturation becomes easier.

**Phenic Acid in Intermittent Fever.**—(Therapeu. Gazette) Having once heard of the advantages of phenic acid in refractory cases of malaria, Dr. Narich resolved to try this drug hypodermically. He dissolved 7 grains of the crytals in two fluidounces of water, and injected a small quantity in the right arm. There appeared redness, with elevation, and a somewhat erysipelatous appearance round the point of injection, but disappeared soon after. A second injection was followed by rather a painful induration which lasted four days. His mode of injection was as follows: On the first day he injected one syringeful. On the four succeeding days six syringes daily: three in the morning and three at night. All in all, thirty-three injections were made. On the fifth day the patient complained of malaise, which increased on the following day, and forced the physician to discontinue the injection on the seventh day. Since the twentieth injection, however, up to date of the publication, for an interval of nine months, the patient has had no more paroxysms.

Although the successful employment in a single case of a certain drug does not suffice to establish its virtues, the phenic acid injections should be borne in mind by those practitioners who have to deal with some very refractory cases of malaria, in which the usual medication has failed.

**Atropine to Prevent Shock After Operation.**—Dr. Stimson stated in N. Y. Medical and Surgical Society that he has been in the habit, in cases which were to undergo grave operations, of employing a small quantity of atropine hypodermically just before beginning the operation. He was led to this use of atropine by knowledge of the fact established by physiologists that it would prevent the inhibitory action upon the heart of efferent pneumogastric currents excited by irritation of important sensory nerves. He has tried it in a number of cases in amounts varying from 1-64 to 1-96 of a grain and he believes it has directly contributed to the success of the operations.

Dr. Stimson found it difficult to speak with precision on such a point, because of the lack of positive evidence, but he could say that patients thus treated had left the table with a better pulse and less collapse or shock, than those who had not been protected by the use of atropine.

**Pilocarpin in Pneumonia.**—(Medical and Surgical Reporter) The Paris correspondent of the British Medical Journal tells us that Dr. Humbert Molliere has successfully treated double pneumonia with pilocarpin; the patient, exhausted by dysentery and albuminuria, was attacked in both lungs, and the intestinal disturbance was greatly aggravated. A centigramme of pilocapin was injected; the respiratory movements fell from forty-eight a minute to twenty-four, and dyspnœa was much relieved; four hours later, a fresh injection was made, and was repeated the next morning; each injection was followed by profuse sweats and salivation, and dyspnœa was greatly relieved. The patient rapidly recovered. In administering pilocarpin, M. Molliere was guided by former experience. An elderly man with uremia accompanied with dyspnœa and delirium, who seemed dying, was greatly relieved, and ultimately cured by a similar treatment. Dr. Molliere describes another case. A young woman with a comatose form of uremia and renal lesion, following a cardiac affection, was freed from the comatose condition by injections of pilocarpin.

**Use of Sulphur in the Treatment of Chronic Dysentery.**—(New York Medical Journal) Schmitjan (quoted in Union Medicale, January 17, 1886) begins the treatment of subacute and chronic dysentery with an emetic of ipecac, and then gives, every three hours a teaspoonful of this powder:

℞. Sublimed and washed sulphur. } aa..............1 part.

Powdered fennel seed, }

Powdered sugar, } aa....................2 parts.

Powdered gum arabic, }

The sulphur, he says, acts in the same way as saline puragtives and calomel act; moreover, it may have peculiar topical action upon the inflamed and ulcerated mucous membrane, comparable to that of the sulphurous waters, purging on the one hand, and having an antiseptic and healing effect on the other.

**Enuresis Nocturna.**—Dr. Kelp (Medicinsche Central-Zeitung) has used hypodermic injections of strychn. nitr. in very intractable cases of enuresis with the best results. He injects about 1-100 to 1-75 gr. into the sacral region, and repeats as soon as it becomes necessary. In many cases in which the unfortunate sufferers were troubled night after night for a long time, the disease was arrested after one injection for some days, when it reappeared, and required another injection, with still better result. He does not exactly state how many injections he made in the various cases, but remarks that he invariably cured the patients with this treatment in a short time. His last case reported was a girl of eighteen years, who had been sick with scarlatina about three months before she came under his care; she had suffered with nocturnal enuresis since she recovered from her sickness, in spite of the best care, tonics, and the avoidance of all sorts of drinks at night, and with the precaution of urinating before going to bed. After the first injection of 1-75 gr. she slept without any disturbance for four nights; on the fifth night she wet herself again, after which injections were repeated every few days, and she was soon relieved entirely of this loathsome disease.

**Water for Infants.**—A physician of the New York Medical Nursery and Child's Hospital, (N. Y. Medical Times) believes, from his practice, that infants generally, whether brought up at the breast, or artificially, are not supplied with sufficient water, the fluid portion of their food being quickly taken up, leaving the solid too thick to be easily digested. In warm, dry weather healthy babies will take water every hour with advantage, and their frequent fretfulness and rise of temperature is often directly due to their not having it. A free supply of water, and restricting the frequency of nursing has been found in the nurserv to be a most effectual check in cases of incipient fever, a diminished rate of mortality and marked reduction in the number of gastric and intestinal complaints being attributed to this cause. In teeth-cutting, water soothes the gums and frequently stops the fretting and restlessness universal in children at this period.

**A New National Association of Physicians** (announced in New York Medical Record) has been decided upon on the following basis :

1. To form an association of physicians and pathologists, of which the number of members shall be limited to one hundred.

2. To hold an annual meeting in the month of June in the city of Washington.

3. To hold the first meeting on Tuesday and Friday, the 16th and 17th of June, 1886, under the presidency of Dr. Francis Delafield, of New York.

The following are the names of the committee who are to select and notify the parties to be invited to join : Francis Minot, Boston, Chairman of the Committee; William H. Draper, New York City; William Pepper, Philadelphia; R. Palmer Howard, Montreal, Canada ; Alfred L. Loomis, New York, City; William H. Welch, Baltimore, Maryland; Francis Delafield, New York City; James Tyson, 1506 Spruce street, Philadelphia, Secretary.

**Manaca in Muscular Rheumatism.**—Exchange: Inasmuch as I can not be with you, I have determined to contribute a short dissertation on the Use of Manaca in Muscular Rheumatism. My experience with this drug has been solely in the form of fluid extract, and is mostly a personal testing, confirmed by observation of its effects on others.

Given, a case of rheumatism with soft skin and absence of feverish symptoms, affecting more especially the muscles, including their tendons, perhaps occasionally affecting the joints ; the pain being dull and heavy, but continuous.

℞. Manaca.................................ʒ j to jss,

Water.................................℥ jv.

Teaspoonful three times a day will give quicker relief than any remedy that I know of.

I can find no positive symptom of its action save that the pain gradually fades out, and that the parts affected gradually assume their natural suppleness and strength.

**Tests for Albumen.**—The New York Medical Times says: Our own observations coincide with those of Dr. Harris in use of the heat test for albumen, it being quite as satisfactory as any other and used with much less trouble. The urine, however, must be just acid enough. If it is alkakine it must be acidulated before boiling with a little acetic acid, being careful not to add too much acid.

**The Interval between Marriage** (British Medical Journal, 1883) and the birth of the first child, is given in over 6,000 cases in a table prepared by Ansell. This gives a mean interval of nearly sixteen months. The majority are born before the close of the first year, nearly seven-eighths before the close of the second year. In 421 cases the first child was born after three years of married life, and before the fourth year was completed, while in the years after the fourth there were only 222 taken altogether. From these data and similar results in other tables, Dr. J. Matthew Duncan concludes that married women delaying the commencement of fertility beyond six months, are already exhibiting a degree of relative sterility, and, that when a married woman remains until the end of the fourth year without conceiving, the probabilities are strong that she will prove absolutely sterile.

**Sulphate of Aniline for Epilepsy.**—We have prescribed *sulphate of analine* in some cases of epilepsey and infantile convulsions, with sufficient benefit to encourage a further trial of this little-used salt. The dose for infants and children under ten years was one grain dissolved in water, with a few drops of diluted sulphuric acid, administered twice or three times a day. Two grains were given to adults. It had no ill effects in these quantities. Sulphate of aniline is a salt of a light grey color, which deepens under the influence of light. It gives a "tarry" odor of coal to the excretions, being itself a product of the distillation of coal. It was formerly employed in the treatment of chorea and some obscure nervous affections, but fell into disuetude. It dilates the pupil. It is soluble in ether, alcohol, and in fixed and volatile oils, and sparingly in water.—*Exchange.*

**Lanolin, or Wool Fat.**—(Medical Press) It is said of this substance that it never becomes rancid, and its smell should remind one of wool.

My experience with the remedy, with that of other physicians, has been but limited, yet I do not hesitate to pronounce the results so far obtained most promising.

The first question which presented itself was, whether the skin would bear it well. From its use in over four hundred cases, in the hospital and private practice of Dr. Lassar, the dermatologist, no irritation of the skin was ever produced—a result which my own experience, during the years in which I have been experimenting with it, confirms. For this reason alone, it is to be highly recommended for massage.

It is true, it is not as smooth as vaseline, but it has the advantage that the skin, after being rubbed dry with a cloth, still remains soft and pliable.

**Bee-stings.**—Dr. E. Pratt says (in Medical and Surgical Reporter) that having used Squibb's Cholera Mixture for the past year as a remedy for bee-stings, I can say it is a specific, instantaneous in its effect if applied within a few minutes after the stinging, preventing soreness and swelling.

**Treatment of Acute Tonsillitis.**—(Medical and Surgical Reporter) Dr. John Brown states in the British Medical Journal, Feb. 6, 1886, that it is a rare event for suppuration to occur in acute tonsillitis, if treated early with the following mixture:

| | | |
|---|---|---|
| ℞. | Sodæ salicyl.. | ʒ iss, |
| | Pot. bicarb.. | ʒ iss, |
| | Tinct. aconiti | *m* xl, |
| | Liq. opii sed | *m* xxx, |
| | Sp. chlorof. | ʒ ij, |
| | Aquæ ad | ℥ viij. |

One ounce to be taken every two or three hours for the first thirty-six hours.

The same mixture is his sheet-anchor for rheumatic fever. He finds that the large doses prescribed in the London hospitals are not needed. Small doses, frequently repeated, are more effective, and almost free from the nauseating effects produced by large doses of salicylate of soda.

**Vomiting in Pregnancy.**—In the St. Louis Courier we read of a young woman, primapara, of feeble constitution, who had vomited from the second month of pregnancy. At the end of the fifth month the vomiting became so violent as to threaten death, there being syncope, absolute prostration of power, noises in the ears, chills, cold and profuse sweats, frequent and filiform pulse, etc. In their turn, antispasmodics have been used (ether, valerian, musk), the opiates, carbonized and iced drinks, iodine externally and internally, blisters upon the epigastrium, hypodermic injections of morphia—in fact, every known means of arresting vomiting—all without avail. It was suggested to try irrigations of ether upon the epigastrium. The effect was instantaneous ; a single irrigation sufficed to cut short the vomiting. The patient drew a few long breaths. said she was cured, and left perfectly well. The vomiting returned twice subsequently, and at each time the ether irrigations arrested the trouble.—*Cincinnati Med. News.*

**Granular Lids.**—In a case of granular lids of long standing Dr. Mitchell (in California Medical Journal) says: Knowing the beneficial influence of hydrastis on the mucous membrane as an astringent, I concluded to give it a trial. I used the specific tincture, full strength ; one drop applied morning and evening with an ordinary dropper. On the evening of the second day the inflammation was considerably reduced and a general improvement was noticeable. In fact, she stated (to use her own words), "I believe, doctor, they are about well."

**To Reduce Hernia.**—(Medical World) It is stated in a foreign exchange that strangulated hernia may be reduced, without opertion, by the simple application of ether continuously over the seat of the obstruction, with the addition of light pressure. Cases of several days' duration are stated to have thus been spontaneously reduced in a few hours.

**Vaginal Pastiles.**—Germicide: Pastiles prepared from boracic acid and cocaine are exceedingly efficacious in absorbing thickening of the neck of the uterus, and fibroid tumors on vaginal walls, healing all kinds of ulcers on the os or neck of the uterus. They have a sort of magical effect in bracing up a devitalized uterus.

One can be inserted thrice daily, and while doing so, they should be pushed up as far as possible.

Before their introduction, the vagina should be well washed out with a fountain syringe charged with a saturated solution of either boroglyceride or boracic acid. They are of great utility in all diseases of the vaginia.

**Chronic Enlargement of Tonsils.**—In answer to Dr. Gaff's inquiry in regard to hypodermic injections in the above named condition, Dr. Beresford, in the October number of the Medical Advocate, says ; "By the use of a strong solution of tanic acid injected two or three times a week, with the daily use of a gargle of the same, the knife need never be resorted to."

I have used the above treatment in the case of a young man, æt. about twenty years, with good success. I make an application of muriate cocaine before inserting the needle, and used the injection every three days.—*California Med Jour.*

**The method** (Chinese Imperial Maritime Customs Reports, 28th issue) employed by the Amis tribe of savages, which inhabit South Formosa, to harden their children might, perhaps, be adopted in this country with advantage. When born, the child is immediately thrown into a tub of cold water, and then taken out and wiped dry. This is continued daily for about a month ; after this it is taken every morning to the sea-beach or river, thrown into the water and allowed to struggle. This practice is pursued both in summer and winter, until the child can walk into the water itself. It is said that the Amis children can usually swim long before they can walk.

**Paraldehyde as an Antidote to Strychnine.**—(Practitioner and News) The result of former investigations, proving that paraldehyde reduces in a marked degree the activity of the spinal cord as a reflex center, has led Dr. Bokai to infer that this drug might do service as an antidote to strychnine. The inference was confirmed by some experiments on frogs, rabbits, and dogs. Certain of these animals were given a less than fatal dose of strychnine. None of the animals died, although some of them received ten times the dose of strychnine usually considered fatal.

**Prevention of Cancer.**—(Medical and Surgical Reporter) People should stop worrying themselves about the fact that a relative had cancer, because it does not affect them. They should live more frugally and take plenty of exercise in the open air, and in short, follow hygienic modes of living, and the danger of cancer is much more remote. The cure may be difficult, but prevention seems to be in the power of each individual.

**Dr. Cotter** writes (in Indiana Medical Gazette) that he has had great satisfaction with the use of glycerin painted over the tongue in fevers. It keeps the tongue moist and removes the sensation of great thirst and discomfort caused by a dry and foul tongue.

**Tsuchiakabi.**—A new drug coming from Japan, called tsuchiakabi, has been proposed as a substitute for cubebs, copaiba and drugs of that class. It is described as the fruit of a kind of orchid, possessing a bitter acid taste, but not an unpleasant flavor. An aqueous extract is used in Japan, and an alcoholic extract has also been prepared.—*Druggists Circular.*

**Action of Coffee on Pruritus.**——Dr. Brown-Sequard reported studies in relation to the action of coffee on pruritus ani and vulvæ. In two cases observed for many years, he had been able to observe a constant agreement between the ingestion of the one and the disappearance of the other ; abstention from coffee caused the pruritus to cease entirely.—*Louisville Med. News.*

**A Little Girl** in Watertown, N. Y., (Medical Quarterly) who was dying of scarlet fever, desired to send a kiss to a little playmate in another town. She kissed the letter, which was sent by mail to the little friend who, wholly unaware of the danger incurred, kissed the letter as a message from her dead friend. In a few days she herself died from scarlet fever, contracted by that kiss.

**Salicylate of Lithia in Rheumatism.**—(Medical Record) This drug acts especially well in those cases in which the fibrous tissues are effected. In the subacute and chronic forms also salicylate of lithia acts more promptly than the sodium salt. The drug should be given in doses aggregating one dram per diem, but when more than seventy-five grains a day are given, toxic symptoms are to be produced. It causes headache and deafness, but never the whistling and ringing sounds in the ears which cause such extreme annoyance to the patient. Sometimes also, though rarely, intestinal colic and diarrhœa result. But all the unpleasant symptoms disappear quickly after the discontinuance of the remedy.

**Quinine in Irritable Stomach.**—John Reid, M. A., in the Australasian M. Gaz., for Dec., proposes the following method of administering quinine in cases of irritable stomach. The sulphate of quinine is dissolved in critic acid (glycerin may be added) and made into an ordinary mixture. Bicarb. soda, more than sufficient to neutralize the citric acid of a dose, is added to a half glass of milk. The citric acid and quinine mixture is then added, while constantly stirring. The effervescing draught, which somewhat resembles koumiss, will be tolerated by the stomach, even when the tongue is red and irritable; the tongue after the draught changing its character.

